# No generally increased risk of cancer after total hip arthroplasty performed due to osteoarthritis

**DOI:** 10.1002/ijc.32711

**Published:** 2019-11-05

**Authors:** Nils P. Hailer, Anne Garland, Max Gordon, Johan Kärrholm, Olof Sköldenberg, Niclas Eriksson, Hans Garmo, Lars Holmberg

**Affiliations:** ^1^ Section of Orthopaedics, Department of Surgical Sciences Uppsala University Hospital Uppsala Sweden; ^2^ Swedish Hip Arthroplasty Register Goteborg Sweden; ^3^ Department of Orthopaedics, Institute of Clinical Sciences The Sahlgrenska Academy, University of Gothenburg Gothenburg Sweden; ^4^ Karolinska Institutet, Department of Clinical Sciences at Danderyd Hospital Division of Orthopaedics Stockholm Sweden; ^5^ Danderyd University Hospital Corporation, Department of Orthopaedics Stockholm Sweden; ^6^ Uppsala Clinical Research Center Uppsala Science Park Uppsala Sweden; ^7^ King's College London, School of Cancer and Pharmaceutical Sciences Translational Oncology & Urology Research (TOUR) London United Kingdom

**Keywords:** total hip replacement, total hip arthroplasty, cancer, nationwide, Sweden

## Abstract

Previous studies on the risk of cancer after total hip arthroplasty (THA) contradict each other, and many are hampered by small cohort sizes, residual confounding, short observation times or a mix of indications underlying the THA procedure. We evaluated the risk of cancer after total hip arthroplasty due to osteoarthritis in a nationwide cohort by comparing cancer incidences in individuals exposed to total hip arthroplasty due to osteoarthritis and in unexposed, sex‐, age‐ and residence matched individuals. To address some previous studies’ shortcomings, information on comorbidity and socioeconomic background were obtained and adjusted for. We included 126,276 patients exposed to a cemented THA between 1992 and 2012, and 555,757 unexposed individuals. Follow‐up started on the day of surgery for exposed individuals and respective date for matched, unexposed individuals, and ended on the day of death, emigration, censuring or December 31st, 2012, whichever came first. The Swedish Hip Arthroplasty Registry (SHAR), the Swedish Cancer Registry, the Swedish National Patient Registry and Statistics Sweden were accessed to obtain information on procedural details of the THA, cancer diagnoses, comorbidities, and socioeconomic background. The primary outcome measure was the occurrence of any cancer after the index date. Exposed individuals had a slightly lower adjusted risk of developing any cancer than unexposed individuals (hazard ratio [HR] 0.97; CI 0.95–0.99). The only cancer with a statistically significant risk increase in exposed individuals was skin melanoma (HR 1.15; CI 1.05–1.24). We attained similar risk estimates in analyses stratified by sex, in individuals with minimum 5 years of follow‐up, in an analysis including individuals with a history of previous cancer, and in patients with cementless THA. In this study on a large and well‐defined population with long follow‐up, we found no increased overall risk of cancer after THA. These reassuring findings could be included in the guidelines on preoperative information given to THA patients.

## Abstract 2

**What's new?**

Some evidence has suggested an increase in cancer associated with total hip arthroplasty, but prior studies have been inconclusive. For this study, the authors recruited 126,276 patients who had received cemented THA and 555,575 age‐ and sex‐matched controls. They limited patients to those whose THA was due to primary osteoarthritis, which generated a relatively homogenous patient population in terms of age and general health. The patients were recruited from the Swedish national THA registry, and cancer incidence was compared between the two groups. They found no increase in cancer risk associated with total hip arthroplasty.

## Introduction

Total hip arthroplasty (THA) restores function and gives pain relief to millions of patients worldwide.^1^ The most common underlying diagnosis is osteoarthritis. Other conditions that motivate THA include avascular necrosis of the femoral head, hip dysplasia, rheumatic disease and femoral neck fracture.^2^ There are basically two ways of fixating THA implants to bone: The initial development of the THA procedure during the late 1950s and early 1960s was based on the fixation of implant to bone by the use of “bone cement” that chemically consists of polymethyl‐methacrylate.^3^ This mode of fixation is still very common in the UK, in northern and central Europe, and in Sweden, cemented fixation is chosen for 60% of THA patients.^2^ In contrast, cementless implants were designed to allow for bone ingrowth, and these implants are thus supposed to achieve biological fixation over time.^4^ Cementless fixation is the predominant mode of fixation in the US, southern Europe and Australia.

THA is one of the most common surgical interventions worldwide, and it is deemed so successful that it was termed “operation of the century” in 2007, referring to the 20th century.^5^ Nonetheless, concerns related to the occurrence of malignant disease have accompanied this procedure from its very start, mainly related to two putative mechanisms: (*i*) In the context of cemented THA the potentially toxic agent polymethyl‐methacrylate is used to anchor implants into host bone, and, (*ii*) metal ions such as cobalt, chromium and nickel are released after THA surgery, both after cemented and cementless fixation.^6^, ^7^, ^8^ Indeed, chromosomal aberrations are described in both bone marrow and peripheral blood of patients with THA implants.^9^, ^10^

Several studies report on small but statistically significant increases in the risk of developing hematological and lymphatic cancers in various populations exposed to arthroplasty,^11^, ^12^, ^13^, ^14^ and a small but statistically significantly increased risk of developing other malignancies such as prostate cancer and melanoma has repeatedly been described.^15^, ^16^, ^17^, ^18^ These findings have however been contradicted by several studies that described no increased cancer risk after THA surgery.^16^, ^19^, ^20^

Many studies are hampered by one or several of the following limitations: Either observation times are rather short,^11^, ^16^, ^19^, ^21^, ^22^ or the studied cohorts are comparatively small,^11^, ^12^, ^15^, ^16^, ^18^, ^20^ or, finally, the cohorts are not well defined—with a conundrum of hip and knee arthroplasties^16^, ^17^ or of underlying indications such as osteoarthritis, femoral neck fracture and rheumatoid arthritis.^14^, ^15^, ^19^, ^20^, ^22^, ^23^, ^24^ The mix of indications is problematic since osteoarthritis populations are younger and healthier than patients who receive an arthroplasty due to fracture or rheumatoid arthritis. Aggregating different implants also complicates the picture, since cemented and cementless fixation differ in their use of polymethyl‐methacrylate and in the alloys used for the implants. Further, resurfacing and other metal‐on‐metal arthroplasties result in much higher exposure to metal ions than traditional metal‐on‐polyethylene THA.^25^, ^26^

Thus, uncertainty remains as to whether insertion of THA is associated with an elevated long‐term risk of developing malignancy. This question recently gained public attention since the novel Medical Devices Regulation issued by the European Commission may require orthopedic implants to bear a label communicating the presence of cobalt if the proposal to classify cobalt as a carcinogenic substance is adopted.

We therefore aimed to evaluate the risk of cancer after exposure to cemented total hip arthroplasty performed due to osteoarthritis with adjustment for the potentially disturbing factors comorbidity and socioeconomic background. Furthermore, it was our aim to expand on previous studies by comparing the cancer incidence in exposed individuals not with a standardized incidence ratio but rather with an age‐ and sex‐matched cohort of unexposed individuals. Based on data from the world's oldest THA registry combined with data from a well‐established national cancer registry, we therefore designed a population‐based study comparing a THA‐exposed to an unexposed cohort. Our primary hypothesis was that exposure to cemented THA is not associated with an increased overall adjusted risk of cancer.

## Materials and Methods

### Study design and study population

**Figure 1:**
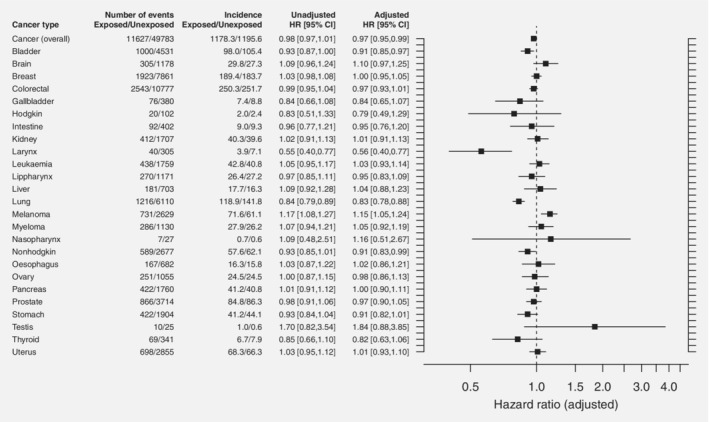
Cancer incidence and risk of cancer in patients exposed to THA compared to unexposed, matched individuals from the general population. “Number of events” gives the number of cancers in exposed and unexposed individuals. “Incidence” describes the cumulative unadjusted cancer incidence per 100,000 person years for exposed and unexposed individuals. “Unadjusted HR” describes the unadjusted hazard ratio for developing cancer in exposed compared to unexposed individuals, and “adjusted HR” describes the hazard ratio for developing cancer in exposed compared to unexposed individuals, adjusted for age, sex, comorbidity, personal income and level of education. The hazard ratios are given with 95% confidence intervals. To the far right, a forest plot illustrates the adjusted risk of cancer in exposed compared to unexposed individuals (hazard ratio = 1) together with 95% confidence intervals.

We performed a nationwide study with an exposed and an unexposed cohort (Fig. 1) by recruiting patients registered in the Swedish Hip Arthroplasty Register (SHAR) who had received a THA due to primary osteoarthritis between 1992 and 2012, matched with unexposed individuals from the general population. Our primary outcome was any occurrence of cancer, secondary outcome measures were specific cancers. In our main analysis, we restricted the exposed population to those who had received cemented THA, thus excluding both cementless and hybrid fixations together with resurfacing arthroplasties, but in an additional sensitivity analysis, individuals who had received a cementless THA were compared to a similarly matched unexposed cohort. The main cohort of patients who had received cemented THA was not subdivided by different cement brands and we also abstained from differentiating by the cup or stem type. All cemented THA contain polymethyl‐methacrylate and there has been no indication that various cement brands would differ in their biological effects, and there is also no biological rationale to assume that different geometric prosthesis designs would confer different mutagenic effects. In the total cohort, 29,930 individuals had been exposed to bilateral cemented THA, and these were included in the main analysis, but separately investigated in a sensitivity analysis.

Each exposed participant was matched to five individuals who were alive and had not been exposed to a THA procedure at the index date (i.e., day of THA surgery for exposed individuals and day of THA surgery of respective case for matched, unexposed individuals). These unexposed individuals were selected from the Swedish population register. Unexposed individuals were matched for age, sex and place of residence. Since matching for age was performed not by exact calendar date but by year of birth, some unexposed individuals had deceased prior to the index date, and these were excluded, but since there was no age restriction in the group of individuals exposed to THA there was also no age exclusion criterion in the group of exposed individuals. Whenever a previously unexposed individual received a THA this individual was censored, but only 0.6% (3,834 individuals) of the initially unexposed population had to be censored due to THA surgery subsequent to the index date.

Information on the occurrence of cancer among exposed and unexposed individuals was obtained from the Swedish Cancer Registry. Occurrence of cancer was defined as the presence of at least one cancer diagnosis code in the Cancer Registry after the index date, and the time to onset of cancer was calculated as the time between the index date and the date at which the first cancer diagnosis was registered. Individuals who had already had a cancer diagnosis at the index date were excluded from the main analyses. In a sensitivity analysis, including individuals with a prior history of cancer, only the occurrence of a novel cancer after the index date was counted as an event, whereas relapses of previous cancers were not counted as events. If an individual suffered from several different cancer diseases or relapses of a primary cancer after the index date, the occurrence of the first cancer diagnosis after the index date was defined as the onset of cancer. Specific cancer forms were defined along with established categories (Supporting Information Table S3).

Follow‐up started on the index date and ended on the day of death, emigration, censuring or December 31st, 2012, whichever came first. Information on death and emigration was collected from Swedish population register. Information on age, sex, Charlson comorbidity Index (CCI)^27^ based on diagnosis codes from the Swedish National Patient Registry (International Classification of Diseases, ICD versions 9 and 10) was obtained to adjust for these relevant confounders. In the Charlson Index, 19 comorbidities are defined by combinations of ICD‐codes, and each comorbidity receives a weighted score that ranges from 1 to 6, depending on the severity of the disease. These numbers are totaled, and a CCI of zero thus implies the absence of registered comorbidities. We categorized the CCI into three levels: (*i*) absence of comorbidities, (*ii*) CCI 1–2 and (*iii*) 3 or more comorbidities.

Socioeconomic status was assessed using personal income and level of education. Personal income was obtained from Statistics Sweden for the year of the index date, and this variable was treated categorically after division along with quartiles. The level of education, again obtained from Statistics Sweden, was separated into four classes: a base category including either no school education, less than 9 years of school or an unknown level of education; followed by the three categories, at least 9 years of school education, high school education and completion of a university degree.

We had no access to data from the Swedish Prescription Register and were thus unable to assess exposure to prescribed drugs.

### Sources of data

The *SHAR* has collected information on all THA procedures performed in Sweden since 1979, it has a completeness of 96‐98% and has been validated repeatedly.^28^, ^29^ *Statistics Sweden* is a registry that collects demographic and socioeconomic information on the entire Swedish population, that is, marital status, level of education and personal and family income. The *Swedish National Patient Register* contains information on diagnosis codes and dates of admissions and discharge for all individuals in Sweden, and the positive predictive value is estimated at around 90 ± 5%.^30^ The *Swedish Cancer Registry* was founded in 1958 and contains information on details including date of diagnosis (clinical or morphological), type of tumor and date and cause of death.^31^, ^32^Based on the 10‐digit personal identification number that all Swedish citizens are assigned at birth or immigration, linkage of information from the registries described above was performed.

### Statistics

Means, medians, ranges and standard deviations described continuous data and categorical data were cross‐tabulated and proportions assessed using the chi‐square test. Cumulative cancer incidence was calculated as the number of incident cancers per 100,000 person‐years for the two respective cohorts. Cox regression models were fitted in order to calculate unadjusted and adjusted hazard ratios (HR) with 95% confidence intervals (CI), with adjustment for relevant confounders (age, sex, CCI, personal income and level of education). The assumption of proportionality of hazards was investigated by plotting unadjusted survival curves for each specific cancer for exposed and unexposed individuals, and no major deviations from this assumption were found. The level of significance was set at *p* < 0.05 in all analyses. The R software (package 3.4.0^33^) was used.

### Ethical approval

Ethical approval for our study was obtained from the Regional Ethical Review Board in Gothenburg (2013: 360‐13). In Sweden, no individual written consent is required for the collection of data into the registries mentioned above. Registration to Statistics Sweden, the Swedish National Patient Register and the Swedish Cancer Register is mandated by law, but use of data for research is regulated by ethical board approval. Individuals are informed that they are included in SHAR, and that the information gathered may be used in research, with the possibility to opt‐out at any time. This is in consistency with Swedish Patient Data Law of 2009 and the Personal Data Act of 1998.

## Results

### Characteristics of the study population

The study population consisted of 126,276 exposed individuals who had received a cemented THA due to osteoarthritis and 555,757 unexposed individuals matched for age, sex and residence. Then, 3,518 individuals in the unexposed cohort later received a THA during their observation time and were censured at their date of surgery. The exposed/unexposed ratio was 1/4.4. The median observation time was 14.6 years for exposed and 14.1 years for unexposed individuals. Mean age at the index date was 71.1 years (range 16–100, standard deviation 8.7).

Individuals in the unexposed cohort were healthier (had fewer registered diagnoses and thus a lower CCI) at the index date than the exposed individuals, with a lower proportion of exposed individuals having a CCI of zero (84.4% *vs*. 89.5%, *p* < 0.001). The socioeconomic status of exposed individuals was slightly better than that of unexposed individuals, with a higher percentage of exposed individuals having completed a high school or university education (48.4% *vs*. 46.9%, *p* < 0.001). A marginally higher proportion of exposed individuals was found among the highest income quarter (22.8% *vs*. 21.6%; *p* < 0.001, Table 1).

**Table 1:** Baseline demographic information on the study population divided by individuals exposed to total hip arthroplasty and nonexposed individuals

	UnexposedNo. 555,757*n* (%)	ExposedNo. 126,276*n* (%)
Sex		
Male	243,725 (43.9%)	53,911 (42.7%)
Female	312,032 (56.1%)	72,365 (57.3%)
Age group		
<50	6,239 (1.1%)	1,324 (1.0%)
50–59	47,166 (8.5%)	10,218 (8.1%)
60–74	296,554 (53.4%)	66,628 (52.8%)
>75	205,798 (37.0%)	48,106 (38.1%)
Charlson index		
0	497,627 (89.5%)	106,556 (84.4%)
1–2	49,679 (8.9%)	17,911 (14.2%)
>2	8,451 (1.5%)	1,809 (1.4%)
Education		
None	18,371 (3.3%)	3,230 (2.6%)
9 years	276,701 (49.8%)	61,994 (49.1%)
High school	178,180 (32.1%)	41,483 (32.9%)
University	82,505 (14.8%)	19,569 (15.5%)
Income		
First quarter	153,142 (27.6%)	33,182 (26.3%)
Second quarter	147,941 (26.6%)	32,611 (25.8%)
Third quarter	134,696 (24.2%)	31,704 (25.1%)
Fourth quarter	119,941 (21.6%)	28,777 (22.8%)
Missing	37 (0.0%)	2 (0.0%)
Cancer before exposure		
No	555,757 (100.0%)	126,276 (100.0%)
Yes	0 (0.0%)	0 (0.0%)

### Cancer after THA

The cumulative, unadjusted cancer incidence was lower in exposed (1,178.3 incident cases per 100,000 person‐years) than in unexposed individuals (1,195.6 incident cases per 100,000 person‐years). Some hematological malignancies such as Hodgkin's and Non‐Hodgkin's lymphoma were less common in exposed individuals, whereas others such as leukemia and myeloma were marginally more common in this cohort (Fig. 1). Some other cancer forms such as cancers of the brain, breast, testis and uterus, and skin melanoma were more frequent among exposed individuals, while lung and larynx cancers were less common among the individuals exposed to a THA.

The unadjusted risk of developing any cancer was slightly lower for exposed compared to unexposed individuals, and in a multivariable model adjusted for age, sex, comorbidity and socioeconomic status we attained a risk estimate that was statistically significantly lower for exposed than for unexposed individuals, with a HR of 0.97 (CI 0.95–0.99) for the exposed individuals (Fig. 1). The adjusted risks of developing the hematopoietic malignancies leukemia, Hodgkin's lymphoma, Non‐Hodgkin's lymphoma and myeloma were not statistically significantly increased for exposed compared to unexposed individuals. The only cancer with a statistically significantly adjusted risk increase in exposed individuals was skin melanoma (HR 1.15; CI 1.05–1.24). The adjusted risk of developing cancer of the testis was higher among exposed individuals, but this estimate did not reach the level of statistical significance (HR 1.84; CI 0.88–3.85). The time to discovery of a melanoma was similar for exposed individuals (8.1 years) as for unexposed (7.8 years), and this was also true for cancer of the testis, with 7.9 years for exposed and 7.6 years for unexposed individuals.

The adjusted risk of developing respiratory tract cancers was lower for exposed individuals, with an adjusted HR of 0.83 (CI 0.78–0.88) for the occurrence of lung cancers in exposed compared to unexposed individuals.

Sex‐stratified models indicated a small but statistically nonsignificant reduction in the adjusted risk of developing any cancer in exposed females (HR 0.99; CI 0.96–1.01), and a slightly more pronounced and statistically significant reduced risk of developing any cancer in exposed males (HR 0.94; CI 0.91–0.97). As described for the unstratified study population, there was no statistically significant increased risk of developing any of the above‐mentioned hematological malignancies in either exposed females or exposed males. In these sex‐stratified models, the only statistically significant risk increase was found for skin melanoma developing in exposed males, with a HR of 1.17 (CI 1.04–1.31; Supporting Information Tables S1 and S2.)

### Sensitivity analyses

Since it is possible that a minimal time of exposure to a THA would be necessary to actually increase the risk of developing cancer, we performed a sensitivity analysis that was restricted to individuals with 5 years of follow‐up. The adjusted HR for developing any cancer in individuals with a minimum of 5 years of follow‐up was 0.98 (CI 0.95–1.01) for exposed compared to unexposed individuals.

A sensitivity analysis included 70,412 individuals with a history of cancer prior to the index date, indicating that 8.8% of the unexposed and 8.6% of the exposed individuals in this larger cohort had had a previous malignancy. In this analysis, the unadjusted and adjusted risk of developing any novel cancer after the index date was again slightly lower for exposed compared to unexposed individuals (data not shown). Subgroup analyses of bilaterally operated patients indicated no increased cancer risk in individuals exposed to a “double dose” of THA (data not shown).

Additional investigations included a sensitivity analysis of 12,845 individuals who had received a cementless THA due to osteoarthritis and 58,411 similarly matched, unexposed individuals. In this cohort, the risk of developing any cancer was again slightly lower for exposed than for unexposed individuals, albeit not statistically significantly (HR 0.92; CI 0.84 ‐ 1.02).

## Discussion

### Principal findings

In this nationwide cohort study, we found that the insertion of a cemented THA due to osteoarthritis is not associated with an overall increased risk of cancer. Previous misgivings about potential increases in the incidence of hemopoietic cancers were not corroborated. We can, however, support the previously described increased risk of developing skin melanomas.

THA is generally considered a very safe procedure, with a low 90‐day mortality around 0.5%,^34^ but concerns related to a potential increase in the risk of developing malignant disease have repeatedly been raised.^11^, ^12^, ^13^, ^14^, ^15^, ^16^, ^17^, ^18^ Due to the topographic proximity of the arthroplasty device to bone marrow induction of malignant disease within the hematopoietic system has been feared, either caused by the compound polymethyl‐methacrylate that is used to obtain stable fixation, or by metal ions. In addition to local effects, systemic effects and malignant transformation at sites distant from the THA could occur, since metal ions are distributed further into parenchymatous organs.^35^, ^36^

### Strengths and limitations of the study

The main strengths of our study are its nationwide matched cohort design, the access to comorbidities and socioeconomic data, and the length of follow‐up. This combination makes the present study unique and hence important since the incidence of cancer is closely associated with both comorbidity and socioeconomic position. Our sources of data are highly valid^28^, ^29^, ^30^, ^32^ and the proportion of missing data in our cohort was low (Table 1).

Limitations to our study are the potential biases at different levels that are common in observational data, and the risk of coding errors, as expected when dealing with patient administrative data. Both THA surgeries and cancer diagnoses must have been missed or erroneously registered, but based on the above‐described validation studies we believe these errors to be small. Importantly, there is no reason to believe that failure to register should be predominantly present in exposed or unexposed individuals.

A specific limitation to our study is detection bias in exposed individuals, who by definition may have been subjected to more frequent contacts with health care providers due to the scheduled preoperative and postoperative appointments related to arthroplasty surgery. In our material, this detection bias may actually be reflected by the slightly higher degree of comorbidities in exposed individuals. This finding is in contrast to previous reports on arthroplasty cohorts that were described as “more healthy” than the general population.^34^ On the other hand, the meantime to detection of cancer was several years after the index date for both exposed and unexposed individuals in our study, indicating that the cancer diagnosis was not linked to diagnostic activity around the time of THA surgery.

### Relation to other studies

Compared with other studies on cancer after joint arthroplasty, one strength of the present study is its large cohort size. Most earlier studies investigating the risk of developing cancer after THA have had considerably smaller cohort sizes (range 433–71,990)^12^, ^14^, ^21^, ^22^ with the exception of Smith's large study comparing metal‐on‐metal with standard articulations.^19^ Some previous studies on cancer after arthroplasty surgery have compared a cohort of patients exposed to a THA with a cohort where no‐one ever was exposed to this procedure, thus resulting in a very theoretical construct. In “real life,” a patient not yet exposed to a THA may well receive a THA at a later time point or develop a cancer while still not exposed to a THA, and our study design simulates a prospective study scenario with inclusion of individuals at baseline without at that point predicting the fate of these individuals.

In our main analysis, we restricted the exposed part of our cohort to patients who had received a cemented THA due to osteoarthritis—thus excluding other modes of fixation and avoiding an unnecessary mix of underlying indications. By only including patients selected for a primary THA due to osteoarthritis we achieved at studying a more homogenous cohort than if patients selected for THA due to hip fracture, hip dysplasia or other indications would have been included; these patients have more diverse baseline characteristics in terms of age and comorbidities. The present study is therefore based on a rather well‐defined sample from the general population. This relatively stringent selection of exposed individuals stands in contrast to several previous studies where uncemented and cemented THA were analyzed together, where THA were not separated from total knee arthroplasties,^16^, ^18^, ^23^ or where patients operated due to rheumatoid arthritis or femoral neck fracture were also included.^11^, ^14^, ^15^, ^20^, ^23^, ^24^ Finally, none of the above‐cited studies adjusted for co‐morbidities and socioeconomic status. A number of previous studies specifically investigated the risk of cancer after resurfacing arthroplasty,^14^, ^19^, ^37^ a type of arthroplasty that we intentionally excluded since these patients are exposed to much higher cobalt and chromium concentrations. However, a very low number of metal‐on‐metal articulations is present in our material since 28 mm metal‐on‐metal bearings were in sporadic use in Sweden during 1992–2012.

Previous studies on the risk of cancer after THA are contradictory since several studies do not replicate the above‐described increased cancer incidence in arthroplasty populations, and some studies specifically contradict the previously described increased frequency of hematological malignancies.^16^, ^19^, ^20^

Our finding of increased melanoma risk in exposed individuals is in accordance with several smaller studies and a meta‐analysis in which a 1.4‐fold risk of developing melanoma was described for arthroplasty patients.^16^, ^17^ It has to be taken into account that melanoma of the skin is a cancer form that is associated with higher socioeconomic status.^38^ Since the exposed individuals in our study were slightly wealthier and had a higher level of education than unexposed individuals their increased risk of developing melanoma may be associated with the exposed individuals’ socioeconomic status that was not fully accounted for by our adjustment, rather than with the exposure to a THA.

Our finding of a statistically nonsignificant but notably increased risk estimate for testicular cancer among the exposed individuals is less obviously the result of selection bias. Testicular cancer incidence varies considerably demographically and geographically, being higher in high‐income countries and regions. The etiology is thought to be governed to a substantial part by environmental factors, for example, exposures in utero and in some occupations.^39^ However, it is less clear if socioeconomic status is a valid proxy for such exposures and if socioeconomic factors within a given country are associated with the risk of testicular cancer. Testicular cancer risk patterns may warrant in‐depth studies based on detailed individual data, and a comparison with cohorts exposed to repeated pelvic radiation for other reasons than the diagnosis and treatment of osteoarthritis would be of great value.

Another potential result of residual confounding is the reduced risk of lung cancer in THA patients, which again may reflect their being more educated and wealthier,^40^ which is associated with less smoking. Since lung cancer is a frequent cancer form, such a bias may falsely reduce the overall cancer risk within the exposed part of our cohort.

## Conclusions

In our study with high statistical precision, we find a barely detectable lower risk of developing cancer in the cohort exposed to arthroplasty surgery. The risk of being diagnosed with melanoma seems higher in arthroplasty patients, but this finding may be due to a failure to fully adjust for socioeconomic confounders. The possibly increased risk of developing testicular cancer may also be related to socioeconomic confounders, but increased exposure to radiation in the context of diagnosing and managing hip osteoarthritis may have contributed to this association—a hypothesis warranting further investigation. Within the studied time frame, we conclude that a THA seems not to confer a clinically relevant risk of developing cancer in a population exposed to THA and this reassuring information may be added to current guidelines on preoperative patient information.

## Supporting information


**Table S1**

**Table S2**

**Table S3**

## Data Availability

The authors declare that data and other items supporting the results in the paper that are minimally required to replicate the outcomes of the study can be made available upon reasonable request.
