# Supplementary material for: No generally increased risk of cancer after total hip arthroplasty performed due to osteoarthritis
Source: Int J Cancer. 2019 Nov 5;147(1):76–83. doi: 10.1002/ijc.32711 (PMC7317978; doi:10.1002/ijc.32711)
Supplement: Supplementary file 1 — Table S1 [file IJC-147-76-s001.doc]

**Supplementary Table 1: Risk of cancers in females exposed to total hip arthroplasty and non-exposed females. Adjustment for age, comorbidities, income and educational level.**

|  | **Unadjusted** | **Adjusted** |  |  |  |
| --- | --- | --- | --- | --- | --- |
| **Variable** | **HR** | **95% CI** |  | **HR** | **95% CI** |
| Cancer | 1.00 | 0.98 - 1.03 |  | 0.99 | 0.96 - 1.01 |
| Bladder | 0.94 | 0.82 - 1.08 |  | 0.90 | 0.79 - 1.04 |
| Brain | 1.16 | 0.98 - 1.38 |  | 1.16 | 0.98 - 1.38 |
| Breast | 1.01 | 0.96 - 1.06 |  | 1.00 | 0.95 - 1.05 |
| Colorectal | 1.00 | 0.94 - 1.07 |  | 0.98 | 0.92 - 1.04 |
| Gallbladder | 0.71 | 0.53 - 0.95 |  | 0.71 | 0.53 - 0.96 |
| Hodgkin | 1.29 | 0.71 - 2.34 |  | 1.24 | 0.68 - 2.26 |
| Intestine | 0.85 | 0.61 - 1.18 |  | 0.84 | 0.60 - 1.17 |
| Kidney | 1.18 | 1.01 - 1.38 |  | 1.17 | 1.00 - 1.37 |
| Larynx | 0.77 | 0.38 - 1.58 |  | 0.75 | 0.37 - 1.54 |
| Leukaemia | 1.02 | 0.87 - 1.19 |  | 1.00 | 0.86 - 1.17 |
| Lipoharynx | 1.01 | 0.83 - 1.24 |  | 0.98 | 0.80 - 1.19 |
| Liver | 0.89 | 0.67 - 1.18 |  | 0.86 | 0.64 - 1.14 |
| Lung | 0.94 | 0.86 - 1.03 |  | 0.93 | 0.85 - 1.02 |
| Melanoma | 1.16 | 1.03 - 1.31 |  | 1.13 | 1.00 - 1.27 |
| Myeloma | 1.15 | 0.95 - 1.38 |  | 1.12 | 0.93 - 1.35 |
| Nasopharynx | 1.36 | 0.37 - 5.03 |  | 1.47 | 0.40 - 5.45 |
| Non-Hodgkin | 0.94 | 0.83 - 1.07 |  | 0.92 | 0.80 - 1.04 |
| Oesophagus | 1.06 | 0.79 - 1.44 |  | 1.06 | 0.78 - 1.43 |
| Ovary | 0.98 | 0.86 - 1.13 |  | 0.98 | 0.86 - 1.13 |
| Pancreas | 1.11 | 0.96 - 1.28 |  | 1.08 | 0.94 - 1.24 |
| Stomach | 0.86 | 0.73 - 1.01 |  | 0.84 | 0.71 - 0.99 |
| Thyroid | 0.77 | 0.56 - 1.05 |  | 0.75 | 0.55 - 1.03 |
| Uterus | 1.01 | 0.93 - 1.10 |  | 1.01 | 0.93 - 1.10 |
