# Supplementary material for: No generally increased risk of cancer after total hip arthroplasty performed due to osteoarthritis
Source: Int J Cancer. 2019 Nov 5;147(1):76–83. doi: 10.1002/ijc.32711 (PMC7317978; doi:10.1002/ijc.32711)
Supplement: Supplementary file 2 — Table S2 [file IJC-147-76-s002.doc]

**Supplementary Table 2: Risk of cancers in males exposed to total hip arthroplasty and non-exposed males. Adjustment for age, comorbidities, income and educational level.**

|  | **Unadjusted** | | **Adjusted** | |
| --- | --- | --- | --- | --- |
| **Variable** | **HR** | **95% CI** | **HR** | **95% CI** |
| Cancer | 0.96 | 0.93 - 0.99 | 0.94 | 0.91 - 0.97 |
| Bladder | 0.94 | 0.87 - 1.02 | 0.91 | 0.84 - 0.99 |
| Brain | 1.02 | 0.85 - 1.23 | 1.04 | 0.86 - 1.25 |
| Colorectal | 0.99 | 0.93 - 1.05 | 0.96 | 0.91 - 1.03 |
| Gallbladder | 1.36 | 0.86 - 2.16 | 1.38 | 0.87 - 2.18 |
| Hodgkin | 0.45 | 0.20 - 1.05 | 0.43 | 0.19 - 1.00 |
| Intestine | 1.09 | 0.80 - 1.49 | 1.09 | 0.80 - 1.49 |
| Kidney | 0.90 | 0.77 - 1.05 | 0.89 | 0.77 - 1.04 |
| Larynx | 0.52 | 0.36 - 0.76 | 0.52 | 0.36 - 0.75 |
| Leukaemia | 1.09 | 0.94 - 1.25 | 1.06 | 0.92 - 1.22 |
| Lipoharynx | 0.95 | 0.80 - 1.14 | 0.93 | 0.78 - 1.12 |
| Liver | 1.23 | 1.01 - 1.50 | 1.16 | 0.95 - 1.41 |
| Lung | 0.77 | 0.70 - 0.83 | 0.75 | 0.69 - 0.82 |
| Melanoma | 1.19 | 1.06 - 1.33 | 1.17 | 1.04 - 1.31 |
| Myeloma | 1.00 | 0.83 - 1.20 | 0.98 | 0.82 - 1.18 |
| Nasopharynx | 0.97 | 0.33 - 2.85 | 1.01 | 0.34 - 2.98 |
| Non-Hodgkin | 0.92 | 0.82 - 1.05 | 0.90 | 0.80 - 1.02 |
| Oesophagus | 1.03 | 0.84 - 1.27 | 1.01 | 0.82 - 1.24 |
| Pancreas | 0.91 | 0.77 - 1.07 | 0.90 | 0.77 - 1.06 |
| Prostate | 0.97 | 0.88 - 1.07 | 0.95 | 0.86 - 1.05 |
| Stomach | 1.00 | 0.87 - 1.15 | 0.97 | 0.84 - 1.11 |
| Testis | 1.75 | 0.84 - 3.63 | 1.84 | 0.88 - 3.85 |
| Thyroid | 1.07 | 0.68 - 1.69 | 1.04 | 0.66 - 1.65 |
