# Supplementary material for: No generally increased risk of cancer after total hip arthroplasty performed due to osteoarthritis
Source: Int J Cancer. 2019 Nov 5;147(1):76–83. doi: 10.1002/ijc.32711 (PMC7317978; doi:10.1002/ijc.32711)
Supplement: Supplementary file 3 — Table S3 [file IJC-147-76-s003.rtf]

Supplementary table 3: Coding of cancer diagnoses.Cancer	ICD7	ICD9	ICD-O/3	
Bladder	181	188·7-9	C67	
Brain, central nervous system	193	171191-2	C47C70-2	
Breast	170	174	C50	
Colon, rectum and anus	153-4	153	C18-21	
Gallbladder	155·1	156	C23·9	
Hodgkin lymphoma	201			
Intestine	152-4	152-4	C17-21	
Kidney	180	189	C64·9	
Larynx	161	161	C32	
Leukaemia	204-7	204-208	C42	
Lip, oral cavity and pharynx	140-8	140-149	C00-14	
Liver	155·0	155	C22	
Lung	162-3	162,165	C33-34C38·4C80·9	
Melanoma	190	172	C44	
Multiple myeloma	203	203	C40, C41	
Nasopharynx	146	147·9	C11	
Non-Hodgkin lymphoma	200, 202	200, 202	C85.9, C44	
Oesophagus	150	150	C15	
Ovary	175·0	183·0	C56·9	
Pancreas	157	157·0-3157·8-9	C25·0-3C25·8-9	
Prostate	177	185·9	C61	
Stomach	151	151	C16	
Testis	178	186·9	C62	
Thyroid	194	193·9	C73	
Uterus	171-2174	180·9182·0-1179·9	C53C54C55	
